# Multimarker Composite Prediction of Early Allograft Dysfunction and 90-Day Mortality After Liver Transplantation: Development and Internal Validation of the DLC Score

**DOI:** 10.3390/jcm15135184

**Published:** 2026-07-02

**Authors:** Jeongjun Lee, Sunyoung Son, Manki Ju

**Affiliations:** Department of Surgery, Gangnam Severance Hospital, Yonsei University College of Medicine, Seoul 06273, Republic of Korea

**Keywords:** delta neutrophil index, lactate clearance, CRP/albumin ratio, early allograft dysfunction, liver transplantation, mortality prediction

## Abstract

**Background/Object:** Early allograft dysfunction (EAD) and 90-day mortality are major challenges after liver transplantation (LT). Combined predictive utility of the delta neutrophil index (DNI), lactate clearance, and C-reactive protein/albumin ratio (CAR) in the post-LT setting remains unexplored. **Methods:** We conducted a single-center retrospective cohort study of 548 adult elective LT recipients (January 2010–December 2023). Serial DNI, lactate clearance, CRP, albumin, bilirubin, and INR were measured during the first postoperative week. Multivariate logistic regression identified MELD score and five postoperative predictors of 90-day mortality: **Results:** DNI ≥ 2.5 at POD 7 (OR 8.42, 95% CI 3.56–19.92), lactate clearance < 15% at 6 h post-reperfusion (OR 4.65), CRP/albumin ratio ≥ 8.5 at POD 3 (OR 3.28), total bilirubin ≥ 10 mg/dL at POD 7 (OR 2.94), and INR ≥ 1.6 at POD 7 (OR 2.18). A five-variable postoperative DLC composite score (0–5 points) achieved an AUC of 0.876 (95% CI 0.831–0.921) for 90-day mortality, significantly outperforming DNI alone (AUC 0.742), MELD (AUC 0.663; both *p* < 0.001), and a bilirubin/INR subset of the Olthoff EAD criteria (AUC 0.784; *p* = 0.014). In a secondary concordance analysis, the DLC score showed concordance with patients meeting EAD criteria (AUC 0.854). Risk stratification produced 90-day mortality of 2.7%, 18.4%, and 55.3% for Low-, Intermediate-, and High-risk groups. **Conclusions:** The DLC score demonstrates promising internal performance for risk stratification within the first postoperative week and warrants prospective external validation prior to clinical adoption.

## 1. Introduction

Liver transplantation (LT) remains the definitive treatment for end-stage liver disease, acute liver failure, and selected hepatic malignancies. Despite advances in surgical technique, organ preservation, and perioperative care, early allograft dysfunction (EAD) occurs in 20–30% of recipients and is strongly associated with prolonged intensive care unit (ICU) stay, sepsis, graft loss, and death [1,2]. The timely identification of patients at high risk for these outcomes is critical to enable targeted therapeutic interventions and optimize resource allocation in the post-transplant period [3].

The Model for End-stage Liver Disease (MELD) score is the primary tool for pre-transplant recipient stratification but has limited utility in the postoperative setting, where dynamic changes in graft function and systemic inflammatory response predominate [4]. Several postoperative biomarkers and pretransplant prediction models—including the recently validated model for EAD after LDLT (MELD, hospitalization status, and graft weight; AUC 0.71 in 2944 patients across 17 centers [5])—have been proposed as individual predictors of adverse outcomes, including hepatic transaminases, bilirubin, INR (reflected in the Olthoff criteria for EAD [1]), lactate clearance (a marker of tissue oxygen utilization and reperfusion adequacy [6,7,8,9]), C-reactive protein (CRP), procalcitonin, and, more recently, the delta neutrophil index (DNI) [10,11,12].

The DNI reflects the fraction of circulating immature granulocytes—a marker of bone marrow compensatory response to systemic inflammation and infection—and is automatically calculated during complete blood count analysis using specific hematology analyzers [13]. Our group previously demonstrated, in a cohort of 393 adult elective LT recipients at our institution (January 2010–December 2018), that a DNI ≥ 2.05% at postoperative day (POD) 14 was an independent predictor of 30-day mortality (OR 31.55, 95% CI 3.83–260.3; sensitivity 81.8%, specificity 82.9%), and exhibited superior discrimination compared with CRP alone [10]. While that study established DNI as a clinically meaningful prognostic marker, several important limitations remained unaddressed: the analysis relied on a single biomarker measured at a single time point (POD 14), the outcome was confined to 30-day mortality without evaluating early allograft dysfunction (EAD) or sepsis, and the prognostic contribution of complementary perioperative markers—including lactate clearance and the CRP/albumin ratio—was not explored.

Lactate clearance—defined as the percentage reduction in serum lactate from baseline over a defined interval—has emerged as an integrative indicator of cardiac output, hepatic perfusion, and the adequacy of reperfusion, offering dynamic prognostic information that complements static biomarkers [6,7,8,9]. C-reactive protein (CRP) is a well-validated marker of systemic inflammation and infection [14], and procalcitonin has demonstrated utility as a diagnostic marker for bacterial infection and sepsis [15]. The CRP/albumin ratio (CAR), combining a positive acute-phase reactant with a negative acute-phase marker of nutritional and synthetic reserve, provides a composite index of systemic inflammation and host physiologic capacity that has demonstrated prognostic superiority over CRP or albumin alone in sepsis, major surgery, and critical illness [16,17,18,19,20].

We therefore hypothesized that integrating DNI, lactate clearance, and the CRP/albumin ratio into a multimarker composite model—the DLC score—would offer improved discrimination for 90-day mortality and clinically relevant postoperative complications, including EAD status, sepsis, and prolonged ICU stay, compared with any single marker or the MELD score. To test this hypothesis, we conducted a large single-center retrospective cohort study using serial biomarker data collected at POD 1, 3, 5, and 7 in adult elective LT recipients. Specifically, the null hypothesis (H_0_) was that the DLC composite score would show no improvement in 90-day mortality discrimination compared with individual biomarkers (DNI, lactate clearance, or CAR) or the MELD score, while the alternative hypothesis (H_1_) was that the DLC composite score would yield significantly superior discrimination.

## 2. Materials and Methods

### 2.1. Study Design and Patient Population

This retrospective cohort study was conducted at Gangnam Severance Hospital, Yonsei University College of Medicine, Seoul, Republic of Korea—a high-volume liver transplant center performing over 60 LTs per year. Clinical, laboratory, and outcome data of all consecutive adult patients (age ≥ 18 years) who underwent primary, elective LT between January 2010 and December 2023 were extracted from a prospectively maintained institutional database. The Korean Network for Organ Sharing (KONOS) annual registry was referenced for national benchmarking [21]. The present cohort (January 2010–December 2023, *n* = 548) encompasses and extends our previously reported cohort (January 2010–December 2018, *n* = 393) [10], adding five further years of prospectively collected data and incorporating serial biomarker measurement and a broader outcome set.

Exclusion criteria were: (1) pediatric recipients (age < 18 years); (2) emergency LT (United Network for Organ Sharing [UNOS] status 1A/1B or equivalent); (3) retransplantation; (4) multi-organ transplantation; (5) incomplete serial biomarker data (defined as missing more than one of the four measurement time points for any key variable); and (6) MELD score > 35 at transplant listing (to minimize confounding from the highest-acuity subgroup, consistent with our prior methodology [10]). The study was approved by the Institutional Review Board of Gangnam Severance Hospital (IRB No. 3-2019-0173), and the requirement for individual informed consent was waived given the retrospective design.

Among the 432 patients excluded by these criteria, the predominant categories were emergency LT status, retransplantation, multi-organ transplantation, and MELD > 35; exclusion for incomplete serial biomarker data constituted a minority of cases. Missingness patterns among the included cohort were limited and judged to be consistent with a missing-at-random mechanism on visual inspection; complete-case analysis was therefore used and multiple imputation was not employed. The exclusion of recipients with MELD > 35 was applied to minimize confounding from the highest-acuity subgroup, in whom outcomes are dominated by pre-transplant physiologic instability and competing risks unrelated to early-postoperative biomarker trajectories; we acknowledge that this introduces spectrum bias and limits generalizability of the DLC score to the sickest transplant candidates (further discussed in Section 4 Limitations).

### 2.2. Biomarker Collection and Definitions

Serial laboratory measurements were obtained at four predefined time points: POD 1 (6–12 h after ICU admission), POD 3, POD 5, and POD 7. Biomarkers collected at each time point included: DNI (%), serum lactate (mmol/L), CRP (mg/L), albumin (g/dL), total bilirubin (mg/dL), INR, platelet count (×10^3^/μL), AST (IU/L), ALT (IU/L), and procalcitonin (ng/mL). Additional data collected included intraoperative peak lactate (mmol/L) and the earliest postoperative lactate at 6 h post-reperfusion.

The DNI was calculated using the ADVIA 2120 Hematology System (Siemens Healthineers, Erlangen, Germany) according to the established formula: DNI = [neutrophil and eosinophil subfraction measured in the myeloperoxidase (MPO) channel] − [polymorphonuclear subfraction measured in the nuclear lobularity channel] [13]. Lactate clearance at 6 h was defined as: LC6h (%) = [(lactate at reperfusion—lactate at 6 h post-reperfusion)/lactate at reperfusion] × 100. Lactate clearance values at POD 1, 3, 5, and 7 were calculated relative to the lactate level at reperfusion using the same formula. Lactate at reperfusion was defined as the arterial lactate concentration measured immediately after portal vein and hepatic artery reperfusion (within 5 min of completion of the vascular anastomoses), prior to closure of the surgical field. The CRP/albumin ratio (CAR) was calculated as CRP (mg/dL; CRP in mg/L divided by 10) divided by albumin (g/dL).

### 2.3. Primary and Secondary Outcomes

The primary outcome was 90-day all-cause mortality after LT. Secondary outcomes were: (1) EAD, defined using the Olthoff criteria (peak AST or ALT > 2000 IU/L within POD 1–7, INR ≥ 1.6 on POD 7, or total bilirubin ≥ 10 mg/dL on POD 7 [1]); (2) post-LT sepsis, defined according to Sepsis-3 consensus criteria (Sequential Organ Failure Assessment [SOFA] score increase ≥ 2 with suspected or confirmed infection [22]); and (3) prolonged ICU stay, defined as ICU duration > 7 days.

### 2.4. Statistical Analysis

Categorical variables are presented as numbers and percentages and were compared using the chi-square test or Fisher’s exact test as appropriate. Continuous variables are presented as mean ± standard deviation or median (interquartile range) and were compared using the Mann–Whitney U test or Student’s *t*-test. Temporal profiles of biomarkers were analyzed using repeated-measures analysis of variance (ANOVA).

Receiver operating characteristic (ROC) curves were generated for each individual biomarker and for the composite model. Optimal cut-off values were determined using the Youden index. Logistic regression analysis was performed to identify independent predictors of 90-day mortality; analyses involving EAD were interpreted as secondary concordance analyses because bilirubin and INR are components of both the DLC score and the Olthoff EAD definition. Candidate variables with *p* < 0.10 in univariate analysis were entered into a backward stepwise multivariate logistic regression model. Variables with *p* < 0.05 in multivariate analysis were retained. The composite DLC score was constructed by assigning one point for each selected postoperative predictor exceeding its optimal threshold and was internally validated using 5-fold cross-validation. Differences in AUC between models were tested using the DeLong method. All analyses were performed using SPSS version 27.0 (IBM Corp., Armonk, NY) and R version 4.3.1 (R Foundation for Statistical Computing). Statistical significance was set at *p* < 0.05.

With 90-day mortality events and a backward stepwise model retaining six variables (MELD plus five postoperative predictors), the events-per-variable (EPV) ratio was approximately 13.7, exceeding the conservative threshold of EPV ≥ 10 recommended for logistic regression stability. A total of 14 candidate predictors entered univariate screening using the *p* < 0.10 threshold for inclusion in the multivariate stage. Optimal thresholds for each retained predictor were derived using the Youden index on the development cohort; because threshold optimization and discrimination estimation were performed in the same dataset, the reported AUCs may be optimistically biased, and 5-fold cross-validation was used to attenuate this optimism. We acknowledge that backward stepwise variable selection can yield unstable models and biased coefficient estimates; shrinkage methods (e.g., LASSO or ridge regression) and bootstrap-based optimism correction are planned for the external-validation phase. The DLC score was assigned equal weights (one point per criterion exceeded) to maximize bedside usability; we recognize that a regression-coefficient-weighted version may improve discriminatory accuracy at the cost of practicability and have noted this as a planned future analysis. Decision-curve analysis and net reclassification improvement, as well as formal calibration assessment (Hosmer–Lemeshow goodness-of-fit, Brier score, and calibration plot), were not conducted in the present development cohort and are pre-specified for the prospective external-validation phase. The reporting of this observational study adhered to the STROBE checklist.

## 3. Results

### 3.1. Patient Characteristics and Outcomes

**Figure 1:**
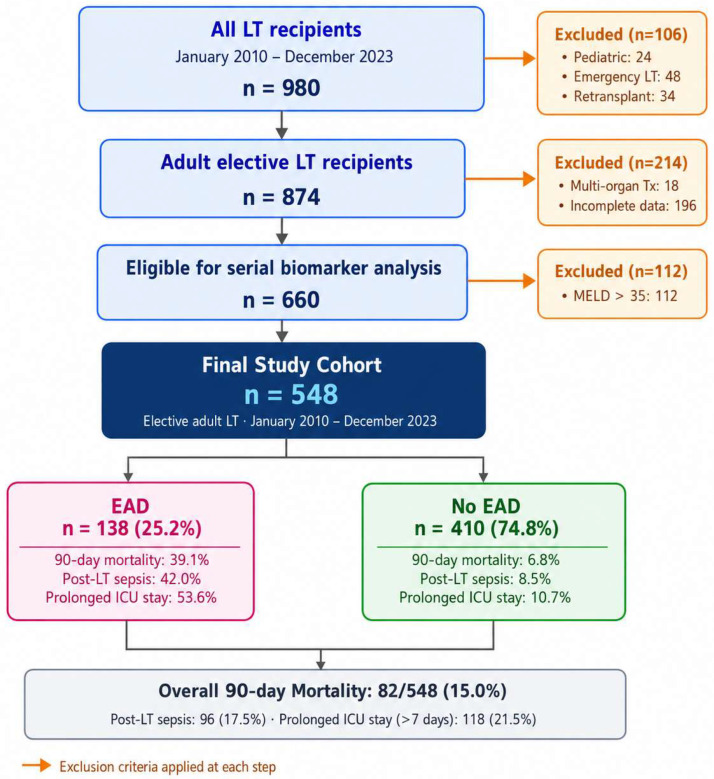
Flowchart of patient selection. Of 980 LT recipients between January 2010 and December 2023, 548 elective adult recipients without emergency, retransplantation, multi-organ transplantation, incomplete-data, or MELD > 35 criteria constituted the final cohort.

Between January 2010 and December 2023, 980 patients underwent LT at our institution. After applying exclusion criteria, 548 elective adult cases constituted the final study cohort (Figure 1). The mean age was 53.8 ± 8.6 years, and 70.8% were male. The mean MELD score was 17.2 ± 9.8. Living donor LT was performed in 386 patients (70.4%). Patient characteristics stratified by EAD status are summarized in Table 1.

EAD occurred in 138 patients (25.2%). Patients with EAD had significantly higher MELD scores (22.6 ± 11.2 vs. 15.4 ± 8.6, *p* < 0.001), longer cold ischemia times (6.4 ± 4.6 h vs. 4.8 ± 3.4 h, *p* = 0.001), and greater intraoperative transfusion requirements (12.4 ± 15.6 vs. 5.8 ± 8.2 units, *p* < 0.001) compared with non-EAD patients. Ninety-day mortality occurred in 82 patients (15.0%). The 90-day mortality rate was significantly higher in the EAD group (39.1%) than in the non-EAD group (6.8%, *p* < 0.001). Post-LT sepsis was diagnosed in 96 patients (17.5%), and prolonged ICU stay (>7 days) was observed in 118 patients (21.5%).

### 3.2. Serial Biomarker Profiles

Table 2 presents the serial biomarker values at POD 1, 3, 5, and 7 stratified by EAD status. EAD patients demonstrated persistently and significantly elevated DNI, lactate, CRP, CAR, bilirubin, INR, AST, ALT, and procalcitonin compared with non-EAD patients at all time points (all *p* < 0.001). Lactate clearance was markedly impaired in the EAD group, with mean LC6h of 12.4 ± 18.6% versus 38.6 ± 18.4% in the non-EAD group (*p* < 0.001). Notably, the CAR showed the most pronounced relative difference between groups at POD 3, where it was approximately 2-fold higher in EAD patients (7.26 ± 4.84 vs. 3.38 ± 2.26, *p* < 0.001). Among EAD patients, DNI demonstrated a rising trajectory from POD 1 through POD 7, in contrast to the declining pattern observed in non-EAD patients, suggesting persistent and escalating systemic granulopoietic stress.

### 3.3. Predictors of 90-Day Mortality: Univariate and Multivariate Analyses

Multivariate logistic regression analysis (Table 3) identified MELD score and five postoperative independent predictors of 90-day mortality: DNI ≥ 2.5 at POD 7 (OR 8.42, 95% CI 3.56–19.92, *p* < 0.001), lactate clearance < 15% at 6 h (OR 4.65, 95% CI 2.01–10.75, *p* < 0.001), CRP/albumin ratio ≥ 8.5 at POD 3 (OR 3.28, 95% CI 1.62–6.63, *p* = 0.001), total bilirubin ≥ 10 mg/dL at POD 7 (OR 2.94, 95% CI 1.38–6.28, *p* = 0.005), and INR ≥ 1.6 at POD 7 (OR 2.18, 95% CI 1.08–4.42, *p* = 0.030). Although MELD score retained independent significance (OR 1.06 per point, 95% CI 1.01–1.12, *p* = 0.018), the DLC score was intentionally constructed as a postoperative bedside score using the five postoperative variables. Age, sex, intraoperative transfusion, CRP level alone, and cold ischemia time did not achieve statistical significance on multivariate analysis.

### 3.4. Development of the DLC Composite Score

**Figure 2:**
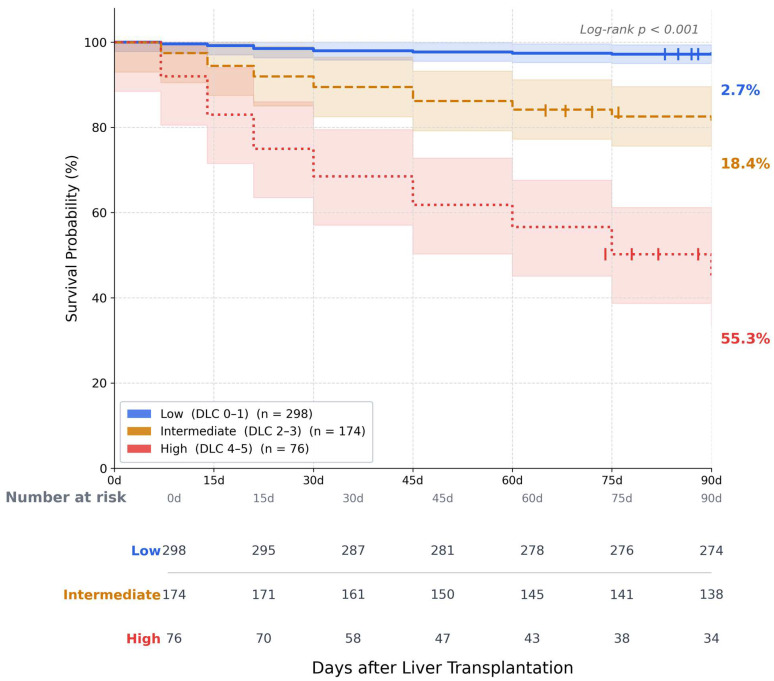
Kaplan–Meier survival curves stratified by DLC score category (Low: 0–1; Intermediate: 2–3; High: 4–5). Log-rank test *p* < 0.001 across all three groups. Numbers at risk reflect the survival analysis set after censoring at each time point.

Based on the five selected postoperative predictors from multivariate analysis, the DLC (DNI–Lactate clearance–CRP/Albumin) composite score was constructed by assigning one point for each of the following: (1) DNI ≥ 2.5 at POD 7; (2) lactate clearance < 15% at 6 h post-reperfusion; (3) CRP/albumin ratio ≥ 8.5 at POD 3; (4) total bilirubin ≥ 10 mg/dL at POD 7; and (5) INR ≥ 1.6 at POD 7. The score ranges from 0 to 5. The CAR threshold was derived for 90-day mortality prediction rather than for discrimination of EAD status. Table 4 presents the risk stratification, with 90-day mortality increasing from 2.7% in the low-risk group (score 0–1, *n* = 298) to 18.4% in the intermediate-risk group (score 2–3, *n* = 174) and 55.3% in the high-risk group (score 4–5, *n* = 76). Kaplan–Meier survival analysis confirmed significant separation between the three risk groups across 90-day follow-up (log-rank *p* < 0.001) (Figure 2).

### 3.5. Discriminatory Performance and Comparison of Models

**Figure 3:**
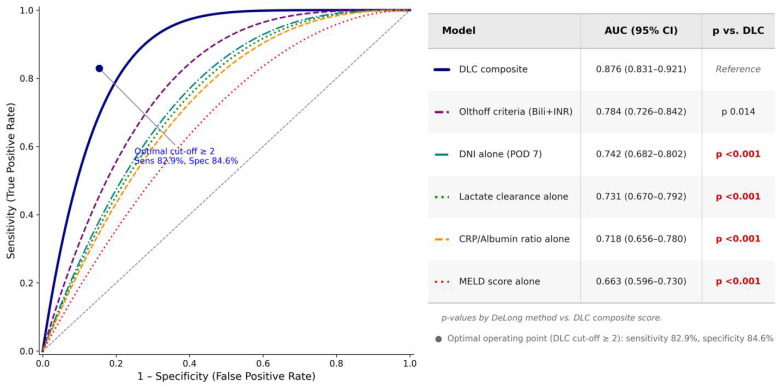
Receiver operating characteristic (ROC) curves comparing the discriminatory performance of the DLC composite score, DNI alone, lactate clearance alone, CRP/albumin ratio alone, MELD score, and the bilirubin/INR subset of the Olthoff EAD criteria for predicting 90-day mortality. AUC values and 95% confidence intervals are shown in the legend.

Table 5 compares the AUC for 90-day mortality across models. The DLC composite score achieved an AUC of 0.876 (95% CI 0.831–0.921), with sensitivity 82.9% and specificity 84.6% at the optimal cut-off of ≥ 2. This was significantly superior to DNI alone (AUC 0.742, *p* < 0.001), lactate clearance alone (AUC 0.731, *p* < 0.001), CRP/albumin ratio alone (AUC 0.718, *p* < 0.001), MELD score (AUC 0.663, *p* < 0.001), and the bilirubin/INR subset of the Olthoff EAD criteria (AUC 0.784, *p* = 0.014). The DLC score also showed concordance with EAD status (AUC 0.854, 95% CI 0.806–0.902); however, this secondary analysis should be interpreted cautiously because bilirubin and INR are included in both the DLC score and the Olthoff EAD definition. Five-fold cross-validation yielded a mean AUC of 0.861 (range 0.842–0.879), confirming internal stability of the composite model. For secondary outcomes, a DLC score ≥ 2 was associated with significantly increased rates of post-LT sepsis and prolonged ICU stay compared with scores 0–1. The ROC curves comparing all models for 90-day mortality discrimination are shown in Figure 3.

## 4. Discussion

In this large single-center retrospective study of 548 elective adult LT recipients, we developed and internally validated the DLC composite score—a five-variable postoperative model incorporating DNI, lactate clearance, CRP/albumin ratio, bilirubin, and INR—that provides significantly superior prediction of 90-day mortality compared with any individual biomarker or MELD score. The key findings of this study are threefold. First, serial measurements at multiple early postoperative time points identified distinct, progressively divergent biomarker trajectories in EAD versus non-EAD patients from POD 1 onwards. Second, three novel or underutilized biomarkers—DNI, lactate clearance, and CAR—emerged as powerful postoperative predictors, complementing conventional hepatic function tests. Third, combining these into a simple integer-based score achieves an AUC of 0.876 for 90-day mortality and enables meaningful stratification into three prognostic risk categories.

The present study directly extends our prior work [10], in which a single DNI measurement at POD 14 (cut-off ≥ 2.05%) achieved 30-day mortality discrimination with a sensitivity of 81.8% and specificity of 82.9% (OR 31.55, 95% CI 3.83–260.3). In the current analysis, incorporating serial DNI measurements from POD 1 through POD 7 into the composite DLC model yielded an AUC of 0.876 for 90-day mortality—substantially exceeding DNI alone at any individual time point (AUC 0.742 at POD 7) and far surpassing the MELD score (AUC 0.663). Beyond mortality, the DLC model showed concordance with EAD status (AUC 0.854), and discriminated post-LT sepsis (AUC 0.847) and prolonged ICU stay > 7 days (AUC 0.823)—outcomes not addressed in the prior study. The EAD analysis should be interpreted as a concordance analysis rather than a fully independent prediction analysis because bilirubin and INR contribute to both the DLC score and the Olthoff EAD definition. The current findings demonstrate that elevated DNI is detectable as early as POD 1 in patients who ultimately develop EAD, and that the DNI trajectory—rising through POD 7 in high-risk patients while falling in those with uncomplicated recovery—provides dynamic prognostic information that a single late measurement cannot capture. The biological rationale is well-established: the DNI reflects emergency granulopoiesis in response to tissue injury, infection, and systemic inflammation [13,23]. In the post-LT context, persistently elevated or rising DNI from POD 1 through POD 7 likely represents a convergence of ischemia–reperfusion injury, early graft dysfunction, and nascent infection—pathologic states that act synergistically to drive mortality [24]. POD 7 was selected as the index time point for DNI, bilirubin, and INR to align with the established Olthoff EAD assessment time point and to capture the latest meaningful measurement within the predefined first-postoperative-week observation window; earlier DNI thresholds may also carry prognostic value and could be examined in future analyses.

Lactate clearance emerged as the second-most potent postoperative predictor (OR 4.65), reflecting the critical importance of hepatic perfusion and oxygen utilization in the immediate post-reperfusion period. Separately, systemic immune–inflammatory markers have been associated with early mortality after liver transplantation, underscoring the prognostic relevance of immune–inflammatory status [25]. Impaired lactate clearance (<15% at 6 h) occurs in the context of low cardiac output, inadequate graft perfusion, or impaired hepatocellular oxidative metabolism—all hallmarks of early graft dysfunction [6,7,8]. Donor characteristics—including graft size, ischemia time, and advanced donor age—also independently modulate early graft function [26]. Golse et al. demonstrated that arterial lactate ≥ 5 mmol/L at the end of LT predicted both primary nonfunction and EAD with an AUC of 0.87 when added to the BAR score [8]. A threshold of 15% clearance differs from the 10% threshold sometimes used in sepsis literature [27], reflecting the distinct metabolic context of reperfusion in LT recipients compared with the general ICU population. The prognostic significance of 6 h lactate clearance also implies that preliminary risk assessment can be initiated within hours of ICU admission, although the complete DLC score requires POD 7 variables.

The CRP/albumin ratio represents a simple, inexpensive, and broadly available composite index that encapsulates both the magnitude of systemic inflammatory response (CRP) and the integrity of host nutritional and synthetic reserve (albumin) [16,17,18]. Its predictive peak at POD 3—with an approximately 2-fold difference between EAD and non-EAD groups—corresponds to the expected zenith of post-operative acute-phase response, following which CAR typically declines in patients with uncomplicated graft function. In patients with EAD or impending graft failure, this decline is attenuated or absent due to persistent hepatic synthetic dysfunction and ongoing inflammatory activation. In this study, CAR was calculated as CRP in mg/dL divided by albumin in g/dL to maintain consistency with the identified threshold of 8.5 [17,18].

The integration of bilirubin and INR within the DLC score is consistent with the Olthoff EAD criteria, confirming that established markers of hepatic synthetic and excretory function retain independent prognostic value for mortality. However, because bilirubin and INR are also part of the Olthoff EAD definition, analyses involving EAD status should be interpreted as concordance analyses rather than fully independent prediction analyses. Their inclusion as two of five equally weighted components—rather than the sole determinants of mortality risk—acknowledges that hepatic graft function is one of several interconnected physiologic processes that collectively determine mortality risk. This holistic model, incorporating innate immune activation (DNI), perfusion and metabolism (lactate clearance), systemic inflammation and nutritional reserve (CAR), and direct hepatocellular function (bilirubin, INR), aligns with the multifactorial nature of post-LT organ dysfunction.

The potential clinical implications of the DLC score, pending external validation, are considerable. Patients with a score of 0–1 (54.4% of the cohort) carry a 90-day mortality of only 2.7% and may be considered for standard monitoring and potential de-escalation of care after clinical assessment. Intermediate-risk patients (score 2–3, 31.8%) with 18.4% mortality warrant intensified surveillance, early consultation for complications, and consideration of prophylactic strategies for sepsis and organ support [28]. High-risk patients (score ≥ 4, 13.9%) with 55.3% mortality represent a cohort in whom aggressive early intervention—including repeat hepatic Doppler ultrasonography, reassessment of immunosuppression dosing, early initiation of antimicrobial therapy, and multidisciplinary discussion regarding retransplantation candidacy—may be warranted. The association of DLC score ≥ 2 with both higher rates of sepsis and prolonged ICU stay further supports its utility in guiding institutional resource planning.

Several limitations of this study must be acknowledged. First, the retrospective design and single-center setting introduce potential for selection bias and limit generalizability; prospective multicenter validation is needed before widespread clinical adoption. Second, our institution transitioned away from the ADVIA 2120 analyzer over the study period, which may have introduced some measurement heterogeneity in DNI values, though we have standardized data using instrument-specific reference intervals. Third, because this is an extended cohort from a single center, changes in surgical technique, immunosuppression protocols, and critical care practice over the 14-year study period represent unmeasured confounders. Fourth, the DLC score as constructed relies on measurements at three different time points (POD 3 for CAR, POD 7 for DNI/bilirubin/INR, and 6 h post-reperfusion for lactate clearance), which means the complete score cannot be computed until POD 7 at the earliest; while this limits its use as an immediate perioperative tool, it is specifically designed as an early warning signal within the first postoperative week to guide subsequent management. Fifth, the current cohort (2010–2023) temporally overlaps with that of our previously published study [10] (2010–2018). Although the present analysis extends the observation window by five years and evaluates a broader range of outcomes and biomarkers, readers should be aware that the 393 patients from the earlier cohort are included within the current 548-patient dataset; independent replication of the DLC model in a non-overlapping or external cohort remains necessary. Sixth, because bilirubin and INR are components of both the DLC score and the Olthoff EAD definition, the reported EAD AUC should be interpreted as concordance with EAD status rather than independent prediction of EAD. Seventh, threshold optimization (Youden index) was performed on the same cohort used for discrimination estimation, which may yield optimistically biased AUC estimates despite 5-fold cross-validation; external validation in an independent cohort is essential for confirming generalizability. Eighth, exclusion of patients with MELD > 35 introduces spectrum bias; the DLC score’s performance in the highest-acuity recipients—who arguably have the greatest clinical need—has not been characterized in this study, and the score should not be extrapolated to this subgroup without further validation. Ninth, backward stepwise variable selection, although widely used, can yield biased coefficients and unstable selection in modest datasets; future analyses incorporating shrinkage methods (e.g., LASSO, ridge regression) may improve coefficient stability and reduce overfitting. Tenth, formal calibration assessment (Hosmer–Lemeshow goodness-of-fit, calibration slope, Brier score, and calibration plot) and clinical-utility metrics (decision-curve analysis, net reclassification improvement) were not performed in the current development cohort and are pre-specified for the prospective external-validation phase. Eleventh, although the analysis is stratified by EAD status, transplant year was not included as an explicit covariate; era-specific changes in surgical technique, ICU protocols, and allocation policy over the 14-year study period may therefore contribute residual confounding, and stratified era analyses are planned for the validation phase.

Future directions include prospective multicenter validation with external calibration of the optimal thresholds, assessment of the DLC score as a guide for intensification of specific interventions, and exploration of a real-time dynamic version of the score using machine learning approaches to incorporate all four time points simultaneously.

## 5. Conclusions

The DLC composite score—integrating DNI, lactate clearance, CRP/albumin ratio, total bilirubin, and INR within the first postoperative week—significantly outperforms individual biomarkers and the MELD score in predicting 90-day mortality after liver transplantation. The score demonstrates promising internal discrimination and enables three-tier risk stratification using routine clinical measurements obtained within the first postoperative week. Given the absence of external validation and the methodological caveats outlined above, the DLC score should be regarded as a candidate tool warranting prospective external validation prior to clinical adoption. Its discrimination of EAD status should be interpreted in light of overlap between DLC components and the Olthoff EAD definition. Prospective multicenter validation studies are warranted to confirm these findings and establish the DLC score as a standard component of post-LT clinical decision-making.

## Figures and Tables

**Table 1:** Baseline characteristics of the study population stratified by early allograft dysfunction (EAD) status.

Variable	Total (*n* = 548)	Non-EAD (*n* = 410)	EAD (*n* = 138)	*p*-Value
Age, years	53.8 ± 8.6	54.0 ± 8.4	53.3 ± 9.1	0.451
Male sex, *n* (%)	388 (70.8)	292 (71.2)	96 (69.6)	0.733
MELD score	17.2 ± 9.8	15.4 ± 8.6	22.6 ± 11.2	<0.001
Donor type, *n* (%)				
Living donor	386 (70.4)	298 (72.7)	88 (63.8)	0.052
Deceased donor	162 (29.6)	112 (27.3)	50 (36.2)	
Cold ischemia time, h	5.2 ± 3.8	4.8 ± 3.4	6.4 ± 4.6	0.001
Operation time, h	11.9 ± 2.3	11.6 ± 2.1	12.8 ± 2.8	0.001
Intraoperative transfusion, units	7.4 ± 10.8	5.8 ± 8.2	12.4 ± 15.6	<0.001
Pre-op DNI (%)	1.42 ± 2.18	1.28 ± 1.94	1.88 ± 2.74	0.032
Pre-op CRP (mg/L)	12.4 ± 24.6	10.8 ± 21.4	17.2 ± 31.6	0.062
Pre-op Albumin (g/dL)	3.2 ± 0.7	3.3 ± 0.6	2.9 ± 0.8	0.001
Pre-op Lactate (mmol/L)	2.6 ± 1.8	2.4 ± 1.6	3.2 ± 2.4	0.002
90-day mortality, *n* (%)	82 (15.0)	28 (6.8)	54 (39.1)	<0.001

Abbreviations: MELD, Model for End-stage Liver Disease; DNI, delta neutrophil index; CRP, C-reactive protein. Data are presented as mean ± standard deviation or n (%). *p*-values represent comparisons between the non-EAD and EAD groups.

**Table 2:** Serial biomarker profiles at POD 1, 3, 5, and 7 stratified by EAD status.

Biomarker	POD 1	POD 3	POD 5	POD 7
Non-EAD Group (*n* = 410)				
DNI (%)	2.84 ± 2.12	2.46 ± 1.98	2.18 ± 1.86	2.12 ± 1.74
Lactate (mmol/L)	2.18 ± 1.42	1.62 ± 1.08	1.38 ± 0.94	1.22 ± 0.82
Lactate clearance (%)	38.6 ± 18.4	52.4 ± 16.8	60.2 ± 15.6	66.8 ± 14.2
CRP (mg/L)	42.6 ± 28.4	86.4 ± 48.6	68.4 ± 42.2	18.2 ± 24.6
Albumin (g/dL)	2.84 ± 0.48	2.68 ± 0.44	2.72 ± 0.46	2.86 ± 0.48
CRP/Albumin ratio	1.56 ± 1.24	3.38 ± 2.26	2.64 ± 1.88	0.68 ± 0.94
Bilirubin (mg/dL)	4.82 ± 3.46	6.24 ± 4.18	6.12 ± 4.42	4.86 ± 3.62
INR	1.62 ± 0.42	1.44 ± 0.36	1.32 ± 0.28	1.24 ± 0.22
AST (IU/L)	842 ± 1246	284 ± 426	148 ± 214	86 ± 124
ALT (IU/L)	648 ± 986	224 ± 348	128 ± 196	74 ± 108
Procalcitonin (ng/mL)	2.84 ± 4.26	1.62 ± 2.84	1.14 ± 1.98	0.82 ± 1.46
EAD Group (*n* = 138)				
DNI (%)	4.28 ± 3.14 *	4.86 ± 3.68 *	5.24 ± 4.12 *	5.82 ± 4.64 *
Lactate (mmol/L)	4.86 ± 2.84 *	3.42 ± 2.14 *	3.08 ± 2.06 *	2.84 ± 1.86 *
Lactate clearance (%)	12.4 ± 18.6 *	28.6 ± 22.4 *	36.4 ± 24.8 *	44.2 ± 26.4 *
CRP (mg/L)	68.4 ± 46.8 *	148.6 ± 84.2 *	126.8 ± 78.4 *	98.6 ± 72.4 *
Albumin (g/dL)	2.42 ± 0.52 *	2.18 ± 0.48 *	2.12 ± 0.46 *	2.08 ± 0.44 *
CRP/Albumin ratio	2.98 ± 2.24 *	7.26 ± 4.84 *	6.24 ± 4.26 *	4.86 ± 3.82 *
Bilirubin (mg/dL)	8.64 ± 6.28 *	12.46 ± 8.62 *	14.28 ± 9.84 *	16.42 ± 11.24 *
INR	2.18 ± 0.68 *	2.06 ± 0.62 *	1.96 ± 0.58 *	1.88 ± 0.54 *
AST (IU/L)	3486 ± 2814 *	1648 ± 1286 *	984 ± 846 *	624 ± 568 *
ALT (IU/L)	2648 ± 2184 *	1284 ± 986 *	824 ± 684 *	528 ± 464 *
Procalcitonin (ng/mL)	8.64 ± 12.46 *	6.28 ± 8.84 *	4.86 ± 6.42 *	4.12 ± 5.86 *

Abbreviations: POD, postoperative day; DNI, delta neutrophil index; CRP, C-reactive protein; INR, international normalized ratio; AST, aspartate aminotransferase; ALT, alanine aminotransferase. CAR was calculated as CRP (mg/dL; CRP in mg/L divided by 10) divided by albumin (g/dL). * *p* < 0.001 vs. non-EAD group. Data are presented as mean ± standard deviation.

**Table 3:** Univariate and multivariate logistic regression analysis for 90-day mortality.

Variable	Univariate OR	*p*	Multivariate OR (95% CI)	*p*-Value
MELD score (per point)	1.08	0.002	1.06 (1.01–1.12)	0.018
DNI ≥ 2.5 at POD 7	8.84	<0.001	8.42 (3.56–19.92)	<0.001
Lactate clearance < 15% at 6 h	5.12	<0.001	4.65 (2.01–10.75)	<0.001
CRP/Albumin ratio ≥ 8.5 at POD 3	3.46	<0.001	3.28 (1.62–6.63)	0.001
Total bilirubin ≥ 10 mg/dL at POD 7	3.08	<0.001	2.94 (1.38–6.28)	0.005
INR ≥ 1.6 at POD 7	2.64	0.001	2.18 (1.08–4.42)	0.030
CRP ≥ 100 mg/L at POD 3	2.84	<0.001	—	0.214
Age	1.02	0.384	—	—
Sex	0.98	0.842	—	—
Intraoperative transfusion	1.04	0.168	—	—
Cold ischemia time ≥ 8 h	2.24	0.012	1.84 (0.88–3.84)	0.104

Abbreviations: OR, odds ratio; CI, confidence interval; DNI, delta neutrophil index; POD, postoperative day; CRP, C-reactive protein; INR, international normalized ratio; MELD, Model for End-stage Liver Disease. “—” indicates variable not retained in final multivariate model.

**Table 4:** DLC composite score: components, risk stratification, and 90-day mortality rates.

DLC Score Components	Points		
DNI ≥ 2.5 at POD 7	1		
Lactate clearance < 15% at 6 h post-reperfusion	1		
CRP/Albumin ratio ≥ 8.5 at POD 3	1		
Total bilirubin ≥ 10 mg/dL at POD 7	1		
INR ≥ 1.6 at POD 7	1		
Risk Stratification by DLC Score			
DLC Score	Risk category	*n* (%)	90-day mortality
0–1	Low	298 (54.4)	2.7% (8/298)
2–3	Intermediate	174 (31.8)	18.4% (32/174)
4–5	High	76 (13.9)	55.3% (42/76)

Abbreviations: DLC, Delta neutrophil index–Lactate clearance–CRP/Albumin ratio composite score; DNI, delta neutrophil index; CRP, C-reactive protein; INR, international normalized ratio; POD, postoperative day.

**Table 5:** Comparison of discriminatory performance (AUC) for 90-day mortality prediction.

Model/Marker	AUC (95% CI)	Sensitivity/Specificity	*p* vs. DLC *
DLC composite model	0.876 (0.831–0.921)	82.9%/84.6%	Reference
DNI alone (POD 7)	0.742 (0.682–0.802)	74.4%/72.8%	<0.001
Lactate clearance alone	0.731 (0.670–0.792)	72.0%/70.4%	<0.001
CRP/Albumin ratio alone	0.718 (0.656–0.780)	70.7%/69.2%	<0.001
MELD score alone	0.663 (0.596–0.730)	62.2%/63.8%	<0.001
Bilirubin + INR (Olthoff EAD criteria subset)	0.784 (0.726–0.842)	78.0%/77.2%	0.014

Abbreviations: AUC, area under the receiver operating characteristic curve; CI, confidence interval; DNI, delta neutrophil index; MELD, Model for End-stage Liver Disease; DLC, composite model. * *p*-values for AUC comparison versus DLC composite model using the DeLong method.

## Data Availability

The data presented in this study are available on reasonable request from the corresponding author. The data are not publicly available due to institutional data sharing agreements and patient privacy regulations.

## Abbreviations

ALT	alanine aminotransferase
ANOVA	analysis of variance
AST	aspartate aminotransferase
AUC	area under the receiver operating characteristic curve
CAR	CRP/albumin ratio, calculated as CRP (mg/dL) divided by albumin (g/dL)
CRP	C-reactive protein
CRRT	continuous renal replacement therapy
DLC	delta neutrophil index–lactate clearance–CRP/albumin composite score
DNI	delta neutrophil index
EAD	early allograft dysfunction
ICU	intensive care unit
INR	international normalized ratio
LC	lactate clearance
LT	liver transplantation
MELD	Model for End-stage Liver Disease
OR	odds ratio
POD	postoperative day
ROC	receiver operating characteristic
SOFA	Sequential Organ Failure Assessment
UNOS	United Network for Organ Sharing
